# When and Why Does Materialism Relate to Employees’ Attitudes and Well-being: The Mediational Role of Need Satisfaction and Need Frustration

**DOI:** 10.3389/fpsyg.2017.01755

**Published:** 2017-10-10

**Authors:** Wenceslao Unanue, Konrad Rempel, Marcos E. Gómez, Anja Van den Broeck

**Affiliations:** ^1^Business School, Universidad Adolfo Ibáñez, Santiago, Chile; ^2^Faculty of Economics and Business, KU Leuven, Leuven, Belgium; ^3^Optentia, North-West University, Vanderbijlpark, South Africa

**Keywords:** materialism, values, need satisfaction and frustration, employees’ attitudes and well-being

## Abstract

Materialistic values may be detrimental for people’s well-being. However, we know little about *why* (i.e., explaining mechanisms) and *when* (i.e., boundary conditions) this is the case. Although low satisfaction of the psychological needs is said to play a key role in this process, a recent meta-analysis indicates that the explaining power of need satisfaction is limited and suggests that need frustration may be more important. Moreover, although materialism may be detrimental in some life domains, studies in materialistic contexts such as work are lacking, particularly in the non-Western world. In response, we put need frustration to the fore and examine both need satisfaction and frustration as the underlying processes in the relation between materialism and employee attitudes and well-being in two Latin–American countries. The Chilean sample (*N* = 742) shows that materialism at work is associated with less positive (work satisfaction and engagement) and more negative (burnout and turnover intentions) outcomes, even when controlling for workers’ income. Notably, need frustration explained the detrimental effects of materialism alongside need satisfaction in a unique manner, showing that it is essential to distinguish both constructs. Results were replicated in Paraguay (*N* = 518) using different positive (organizational commitment and meaning at work) and negative (negative emotions and job insecurity) outcomes, adding to the generalizability of our results across samples of different nations.

## Introduction

Materialism refers to the “importance ascribed to the ownership and acquisition of material goods in achieving major life goals” (Richins, 2004, p. 210). Meta-analytic results show that materialism is associated with lower self-esteem, lower well-being and health and more risk behaviors (Dittmar et al., 2014). Despite this body of evidence, however, we still know little about *why* (i.e., explaining mechanisms) and *when* (i.e., boundary conditions) materialism has such a negative impact.

The underlying psychological processes explaining the negative link between materialism and well-being are not yet well-understood (Dittmar et al., 2014). Self-Determination Theory (SDT; Deci and Ryan, 2000) posits that low satisfaction of the basic psychological needs for autonomy, competence, and relatedness may explain the adverse impact of materialistic values. According to SDT, materialistic strivings would prevent people to function volitionally (autonomy), feel efficacious (competence) and build meaningful bonds with other people (relatedness), and therefore cause stress. Results of a recent meta-analysis indicate that need satisfaction indeed plays a role (Dittmar et al., 2014), but its explaining power is relatively limited, particularly with respect to ill-being (Unanue et al., 2014). To further understand the adverse impact of materialism, we put the construct of need frustration to the fore. Rather than representing low need satisfaction, need frustration captures the degree to which the basic psychological needs for autonomy, competence, and relatedness are actively thwarted for example because people feel pressured, ineffective, or rejected (Bartholomew et al., 2011). Although SDT has mainly focused on need satisfaction, interest in the construct of need frustration is growing (e.g., Landry et al., 2016). However, much in line with recent calls for the study of need frustration in the context of work (Van den Broeck et al., 2016), we are the first to model both need satisfaction and frustration as the underlying processes explaining why materialism may impact positive and negative aspects of employee well-being and their attitudes toward work.

In addition to studying *why* materialism may have negative implications, we also aim to advance our understanding of *when* this is the case. Specifically, conflicting hypothesis exist about the conditions when materialist values are most negative and when they are not so detrimental. On the one hand, following SDT’s assumption that materialism prevents the satisfaction of one’s basic psychological needs or may actively thwart these needs, the adverse impact of materialistic strivings should be universal (Deci and Ryan, 2000). On the other hand, however, several other perspectives suggest that this may not be the case in the context of work, in poorer nations and when taking people’s income into consideration, as in such context materialism may get a different meaning (Dittmar et al., 2014). More specifically, rather than being detrimental, holding materialistic strivings may be motivating in these context, for several reasons. First, they allow people to fit into the work context, which is also capitalistic in nature (Kristof-Brown et al., 2005). Second, rather than a means to boost one’s self-esteem, in poorer nations materialistic values may primary be a mean to provide for oneself and others (Maslow, 1943). Third, rather than being too ambitious, they may represent achievable goals when one has a high income (Latham and Locke, 1991). We aim to further examine this issue.

In short, the current study aims to extend the literature on materialism and SDT by furthering our understanding of *why* and *when* materialistic strivings are detrimental. We examine whether need frustration – in addition to need satisfaction—may help to understand the impact of materialistic strivings, and provide a strong test of SDT’s assumption about the detrimental effect of materialism by studying materialism and its underlying processes in the context of work, in less developed nations, and when controlling for employee income. We particularly selected employees in Chile and Paraguay to participate in this study, as these countries are clearly poorer compared to the average OECD countries. In the following we first describe the relationships of materialism with employee attitudes and well-being, before tapping into the construct of need frustration as an additional explanatory mechanism in these relationships.

### Materialism and Attitudes and Well-being

Materialism has been operationalized in various ways. The most influential approach based on SDT suggests that materialism is a *value* orientation that places strong emphasis on material reward as a pathway to happiness and well-being (Kasser and Ryan, 1996). In line with this approach, materialism is often assessed through the relative importance attached to extrinsic (e.g., money, fame, and image) versus intrinsic values (e.g., personal growth, relationships, community involvement, and physical health). In the organizational arena, for example, attaching importance to extrinsic work values reflects striving for traditional success indicators such as high income (money), prestige (fame) and status (image), while pursuing intrinsic values reflects “employees’ natural desire to actualize oneself, develop and grow at the work place (i.e., self-development), to build meaningful and satisfying relationships with colleagues (i.e., affiliation), and to help people in need (i.e., community contribution)” (Vansteenkiste et al., 2007, p. 253).

The literature on materialism thus focuses on a particular subset of values described in the literature. The differentiation between intrinsic and extrinsic values is for example closely linked to the differentiation between self-transcendence and self-enhancement values in the renowned framework of Schwartz (2012). While self-transcendence values center around to benevolence and universalism, self-enhancement values include mostly the values achievement and power. According to Schwartz values can be defined as “desirable states, objects, goals or behaviors, transcending specific situations and applied as normative standards to judge and to choose among alternative modes of behavior” (Schwartz, 1992, p. 2). Values thus allow to understand whether people are oriented toward themselves (i.e., self-enhancement) or others (self-transcendence) and whether they are anxious and aim to avoid loss (i.e., self-enhancement) or rather anxiety-free and oriented toward self-expansion and growth (i.e., self-transcendence). prevention-focus. Whereas Schwartz assumes that the pursuit of each of these values may have its benefits and pitfalls, SDT advocates that pursuing some values may be more beneficial than others. Specifically, according to SDT, the strivings for intrinsic values aligns with people’s natural inclination for growth and development. Extrinsic values, in contrast, leads people to forsake their inherent growth oriented nature and engage in stressful interpersonal comparisons in which one’s self-esteem is at stage (Kernis, 2003). Materialistic strivings (i.e., valuing extrinsic over intrinsic values) therefore has detrimental consequences.

In line with SDT’s reasoning, meta-analytic findings have convincingly shown that materialistic people tend to show lower well-being in terms of vitality, positive affect, and life satisfaction, but more negative affect, depressive symptoms and anxiety, and more health problems. Materialists have lower self-esteem and engage in more risk behaviors such as drinking, smoking, and drug use (Dittmar et al., 2014) and damage the environment (Unanue et al., 2016). Research attesting to the negative effects of materialism has mainly focused on what people value in life in general or in specific life domains, such as sports (e.g., Vansteenkiste et al., 2004b), education (e.g., Vansteenkiste et al., 2004a), and family (e.g., Promislo et al., 2010). Studies in the domain of work are sparse (Dittmar et al., 2014).

The fact that research on materialistic work values is still in its infancy is surprising, as empirical and theoretical work suggests that the work context may provide a strong test for the assumption that materialism may be detrimental. The meta-analytic findings of Dittmar et al. (2014) suggest that materialistic contexts may attenuate the negative effects of holding materialistic values as an individual. For example, students in business, economics, or marketing may suffer less from materialistic strivings as the person-environment fit theory assumes that people feel well when their values fit the values of the context (Kristof-Brown et al., 2005). As capitalism and associated importance attached to materialism is in the DNA of many organizations, and relationships in the context of work may be more transactional, this raises the question whether having materialist *work* values would also have univocally negative implications for *employee* well-being and attitudes, as would be suggested by SDT.

Some studies seem to suggest that materialistic strivings at work associates with lower work engagement (Schreurs et al., 2014) and less job and career satisfaction (Deckop et al., 2010), higher emotional exhaustion and turn-over intention (Vansteenkiste et al., 2007), less citizenship behavior and more workplace deviance (Deckop et al., 2015).

However, testing the generalizability of these results is still imperative, given the diverging views on whether the previous results on materialistic work values can be generalized to less developed and poorer nations. The sparse studies linking materialism to employee well-being and attitudes have been limited to Western countries such as Belgium (Vansteenkiste et al., 2007), The Netherlands (Schreurs et al., 2014), and the United States (Deckop et al., 2010, 2015). Although SDT would suggest that the negative implications of materialism are universal, based on a Maslowian perspective, strivings for materialistic reward may not be as negative in poorer nations. In such countries, materialism would be highly adaptive to meet physiological needs and to sustain oneself and one’s family (Maslow, 1943), while in more developed and richer countries, most people would already have satisfied these needs and further strivings for material success would be more detrimental (Dittmar et al., 2014).

Taking into account that some suggest that the effects of materialistic strivings may depend on the context (Nickerson et al., 2003), it also becomes imperative to take one’s personal context into account. Following the assumptions of goal setting theory (Latham and Locke, 1991), striving for materialistic values may not be as negative for work related outcomes when employees are able to attain such values, for example when they earn a high income. Although some counter-evidence exist (Vansteenkiste et al., 2007), the debate on the possible attenuating effect of income on the relationship between materialistic values and well-being is still ongoing (Leana and Meuris, 2015). Against this background, we aim to provide a strong test of the impact of materialism by studying the effect of materialistic work values on employee well-being and attitudes within a Latin–American context, while taking into account employee income. Despite the alternative points of view, we follow the dominant theoretical approach to materialism in terms of SDT (Kasser and Ryan, 1996; Deci and Ryan, 2000), the meta-analytic findings of Dittmar et al. (2014) and the first empirical findings in the context of work, and hypothesize that – across nations and different levels of people’s income:

Hypothesis 1: Materialism is negatively associated with employees’ positive attitudes and well-being and positively associated with employees’ negative attitudes and ill-being.

### Self-determination Theory and the Mediational Role of Need Satisfaction

Despite earlier research, exactly why materialism prevents well-being and the development of positive attitudes is poorly understood. According to SDT, the negative implications of materialistic strivings can be explained by the basic psychological needs (Deci and Ryan, 2000). SDT assumes that humans have three innate psychological needs, which are defined as “those nutriments that must be procured by a living entity to maintain its growth, integrity, and health” and are essential for on-going growth, development, integration, and well-being (Ryan and Deci, 2000, p. 326). Three needs are deemed to be essential: the needs for autonomy (volitional functioning), competence (being effective), and relatedness (developing meaningful bonds with others). Several studies have given empirical support to the postulate that satisfaction of the basic needs allows employees to feel well, develop positive attitudes and perform well (see Van den Broeck et al., 2016 for a meta-analysis).

According to SDT, the pursuit of materialistic values detracts people from engaging in need satisfying activities (Vansteenkiste et al., 2007). Higher materialism would thus be associated with lower satisfaction of the psychological needs, which in turns would lead to lower well-being. In the work context for example, employees attaching a high importance to materialistic values are less likely to freely choose those activities they enjoy more, feel more capable of, and which allow them to build intimate and deep human connections with others. In short, materialistic strivings prevents employees from satisfying their needs for autonomy, competence, and relatedness, respectively (Deci and Ryan, 2000; Schreurs et al., 2014). Need satisfaction is hence expected to explain the negative impact of materialism.

Several studies have explored the mediating role of need satisfaction in the relationship between materialism and individual well-being and meta-analytic results tend support to this assumption (Dittmar et al., 2014). However, the explaining power of need satisfaction seems relatively limited, which has lead scholars to call for more research on the role of need frustration as this may capture much better the negative aspects of employee functioning (Unanue et al., 2014; Van den Broeck et al., 2016).

Need frustration is experienced when basic psychological needs are actively thwarted. Similar to the difference between positive and negative affect, need frustration and need satisfaction are thus not just each other’s’ opposites: while low scores on measures of need satisfaction reflect that activities not really tap into the basic needs, need frustration or thwarting implies that the basic needs are actively and intensity violated (Bartholomew et al., 2011). In case of autonomy frustration, people feel pressured, while in case of competence frustration they feel incompetent and worthless, and in case of relatedness frustration they feel rejected. To illustrate, an employee may feel little related to his boss, and thus feel less happy or satisfied. But he could also feel actively rejected, in which case we may suffer from depression or anxiety. Need satisfaction and frustration are not only conceptually different. Further adding to their differentiation, they also have different consequences: while need satisfaction more strongly relates with well-being outcomes such as vitality and life satisfaction, need frustration is said to holds stronger relations with ill-being indicators such as depression (Vansteenkiste and Ryan, 2013; Van den Broeck et al., 2016), and this seems to be supported in empirical research (Bartholomew et al., 2011; Unanue et al., 2014; Chen et al., 2015; Cheon et al., 2015).

Despite the increasing calls to complement the study on need satisfaction with need frustration, to date, research on need frustration in the work context is scarce (Trépanier et al., 2016). Only one published paper provided evidence for the different roles of need satisfaction and need frustration in the link between materialism and well-being outcomes, but studied these relations only in general life settings (Unanue et al., 2014). The current study aims to add to this line of work by examining need satisfaction and need frustration as the underlying processes in the relation of materialistic values with well-being and attitudes in the context of work, in poorer nations and when taking one’s income into account. As mentioned above, some theoretical perspective would suggest that such context may alter the meaning of materialism and – hence – challenge the assumption that materialism may frustrate one’s basic needs. However, building on SDT, we hypothesize:

Hypothesis 2: The association between materialism and attitudes and well-being is explained by both psychological need satisfaction and psychological need frustration at work.

As mentioned previously, recent considerations from SDT have theorized that whereas need satisfaction better captures growth and well-being, need frustration better captures malfunctioning and ill-being (Vansteenkiste and Ryan, 2013). Thus, in the work context, we may expect need satisfaction to be more strongly related to positive job outcomes and need frustration to be more stronger related to negative job outcomes. Further, we hypothesize:

Hypothesis 3: Need satisfaction would primarily explain the associations of materialism with positive attitudes and well-being, whereas need frustration would primarily explain the associations of materialism with negative attitudes and ill-being.

## Materials and Methods

### Participants and Procedure

Study 1 (Chile) and Study 2 (Paraguay) were carried out in accordance with the guideline recommendations of the American Psychological Association, British Psychological Society and World Medical Association Declaration of Helsinki. All subjects gave written informed consent, and the protocol followed the recommendations by the Ethics Committee of a University in Santiago.

We selected full-time working adults from the financial sector (Chile) and from the retail sector (Paraguay), as recent data and research has shown that the financial and retail sectors are among the highest risks of mental health problems (Mutual de Seguridad, 2015; Unanue et al., 2017). Chile and Paraguay are well-suited to test the impact of materialism in poorer nations: whereas the annual GDP per capita (a proxy of labor income) is US$ 39.033 in the average OECD countries, in Chile it is only US$22.128 and US$8.943 in Paraguay, indicating that these countries are relatively poor (using power parity purchase; World Bank, 2014). Moreover, Chilean average labor income represents only 57% on the average OECD income, while Paraguay average income represents 23% (OECD, 2016). Thus, both countries are clearly poorer compared to the average OECD countries, which allow us to test previous finding mostly done in the Western world in a poorer Latin–American context.

In Chile, data was collected with the help of a consultation firm that agreed sending e-mails to employees of its financial client institution. In total, we surveyed the 36 company units spread over all the 15 geographic regions of the country. Respondents were recruited online and were informed of the purpose of the research and invited to participate voluntarily and anonymously.

Participants were sent an introductory email containing a brief description of the study along with a web link to the survey administrated only by the research team. Employees were informed that only the researchers would have access to their data and were allowed to answer the survey at the location of their choosing (e.g., home, office, etc.). These procedure increases the chance that participant’s self-reports could be trusted as representing the facts of the situation or genuine sentiments of the respondents. The sample consisted of 742 employees from the financial sector (approximately 75% of the whole company) working in different positions such as cashiers (62%), managers and supervisors (20%), administrative staff (6%), guards (11%), and others (1%). Participants ranged in age from 18 to 62 years (Mean = 31.80; *SD* = 7.57), 73.0% of the sample was female.

In Paraguay, we followed the same procedure as in Chile. We sampled 518 adult employees from the retail sector (approximately 80% of the whole company), working in a wide array of functions and positions such as managers and supervisors (33%) and administrative staff and sale force (67%) in all of the approximately 120 branches of the company, distributed across the whole country. Participants ranged in age from 18 to 55 years (Mean = 28.18; *SD* = 5.67), 48.0% of the sample was female.

### Measures

Participants from both Chile (Study 1) and Paraguay (Study 2) answered questions about materialism, need satisfaction and need frustration. However, we assessed different aspects of their well-being and work attitudes. Whereas in Chile we included measures of engagement, work satisfaction, turn over intentions and burnout, in Paraguay we analyzed measures of organizational commitment, meaning at work, job satisfaction, negative emotions, job insecurity, and burnout. Study 2 aimed to replicate Study 1 results, using different job outcome measures to expand the validity of the results across different outcome variables, while having positive and negative constructs in each country. Equivalence of meaning from Spanish to English was checked following standard back-translation procedures (Brislin, 1970).

*Materialism at work* was captured with a short version of the Aspiration Index (Kasser and Ryan, 1996) adapted to the context of work (Van den Broeck et al., 2015) in both Chile and Paraguay^1^. Respondents were asked to rate how important it was for them to be “financially successful” (money); “admired for your prestigious position” (fame); “look physically attractive” (image) for the extrinsic values and how important it was for them they could “develop yourself” (self-development); “build good relationships” (affiliation); and “contribute to society” (community involvement) for the intrinsic values. Respondents answered on a five-point scale, from “completely disagree” (1) to “completely agree” (5). Cronbach’s alpha was good in Chile (α = 0.75) and in Paraguay (α = 0.70).

*Need satisfaction and need frustration* at work were measured using a balanced measure including need satisfaction and need frustration in both Chile and Paraguay (Sheldon and Gunz, 2009). We adapted the original scale to the work context. Examples items are “I felt a sense of contact with people I work with” (relatedness satisfaction); “At my work I was successfully completing difficult tasks and projects” (competence satisfaction); “At my work my choices expressed my ‘true self”’ (autonomy satisfaction); “At my work I was lonely” (relatedness frustration); “At my work I experienced some kind of failure, or was unable to do well at something” (competence frustration) and “At my work there were people telling me what I had to do” (autonomy frustration). Respondents answered on a seven-point scale, from “not true at all” (1) to “completely true” (7). The need satisfaction (Chile: α = 0.82; Paraguay: α = 0.77) and need frustration (Chile: α = 0.77; Paraguay: α = 0.73) scales showed good reliabilities.

*Work engagement* was measured in Chile using the subscales of vigor and dedication of the Utrecht Work Engagement Scale (Schaufeli et al., 2006). Example items are “At my work, I feel bursting with energy” (vigor) and “My job inspires me” (dedication). Participants answered on a seven-point scale ranging from never (0) to always (6). The scale showed excellent reliability (α = 0.90).

*Work satisfaction* was assessed in Chile using a single face valid question: “All in all, how satisfied are you with your job?” Participants answered on an 11-point scale, ranging from “extremely unsatisfied” (0) to “extremely satisfied” (10). Previous research and meta-analytic analysis demonstrated that this single question strongly relates to a multi-item measure of work satisfaction (Wanous et al., 1997), suggesting that a single item is sufficient (Dolbier et al., 2005). In Paraguay, work satisfaction was captured asking participants rate from 0 (“extremely unsatisfied”) to 10 (“extremely satisfied”) how satisfied are they with their *salaries*; with the *balance between life and work*; and with the *physical conditions for working*. The scale showed an acceptable reliability (α = 0.68).

*Burnout* was measured in Chile and Paraguay using the Maslach Burnout Inventory-General Survey (MBI-GS; Schaufeli et al., 1996), which includes the subscales of emotional exhaustion (e.g., “*I feel totally exhausted on my job*”) and depersonalization (e.g., “*I doubt the significance of my work*”). Respondents answered on a seven-point scale, from “never” (0) to “always” (6). The scale showed excellent reliability in Chile (α = 0.90) and in Paraguay (α = 0.85).

Employee’s *intention to turnover* was captured in Chile with a single-item question ranging from 0 to 10: “*To what extent have you want to quit your job*?” Meta-analytic findings showed that single-item questionnaires are a comparatively good way to assess the construct (Tett and Meyer, 1993).

*Organizational commitment* was assessed in Paraguay with the measure developed by Cook and Wall (1980). Example item is “*I am quite proud to be able to tell people for whom I work.*” Participants answered on a five-point scale, from “completely disagree” (1) to “completely agree” (5). The internal reliability of the scale was lower than other measures but still acceptable (α = 0.68).

*Meaning at work* was captured in Paraguay using three questions developed by Butler and Kern (2016). An example item is *“In general, to what extent do you feel that what you do in your life is valuable and worthwhile?”* Participants answered on an 11-point scale, from 0 to 10. The scale showed a good reliability (α = 0.81).

*Negative emotions* were captured in Paraguay using three questions developed by Butler and Kern (2016). Participants indicated how often do you feel “*sad*,” “*anxious*,” and “*angry*” at work on an 11-point scale, from 0 to 10. The scale showed an acceptable reliability (α = 0.61).

*Job insecurity* was measured in Paraguay through a single item. Participants rate in a scale from 0 to 10 to what extend they think they *“could be fired”* during the following months.

We also collected data about employees’ income in Chile. The company provided objective measures of salaries.

*Control variables.* In order to control for possible social response biases, we created an index of impression management in both countries by using the social desirability measure developed by Fischer and Fick (1993). Participants answered “true” or “false” to nine questions (e.g., “I have never been irked when people expressed ideas very different from my own”). We also used gender (0 = female; 1 = male) and age as control variables.

### Analytic Strategy

To test our hypotheses, most constructs were modeled using latent variables to control for measurement error (Finkel, 1995), except for work satisfaction and intentions to turnover (Chilean sample) and job insecurity (Paraguayan sample), which were measured through single-item questions and treated as observed variables. Materialism was modeled as a latent variable following a standard procedure to control for response bias (Duriez et al., 2007). First, an overall mean score was calculated. Second, the overall mean score was subtracted from each individual score. Third, the three intrinsic items were reversed. Fourth, we built three parcels using one extrinsic and one reversed intrinsic life goal. Positive scores reflect higher materialism. Following Little et al. (2002), we also created parcels for need satisfaction and frustration, work engagement, burnout, work satisfaction (Paraguayan sample), meaning at work and negative emotions using latent variables. We conducted structural equation modeling using AMOS 22 software. Descriptive statistics and inter-correlations for the study variables are shown in **Table 1**. Following the recommendations of Hu and Bentler (1999) and Kline (2011), we used the chi-square statistic and two fit indices that are relatively free of sample size effects: the comparative fit index (CFI) and the root mean square error of approximation (RMSEA). Values of RMSEA <0.06 (<0.08), and CFI >0.95 (>0.90) were interpreted as evidence of good (acceptable) fit. In some cases, we also used AIC to compare non-nested models.

**Table 1:** Descriptives and inter-correlations between all study variables in Chile and Paraguay.

	*M*	*SD*	*1*	*2*	*3*	*4*	*5*	*6*	*7*	*8*	*9*	*10*	*11*
**Descriptives and inter-correlations between all study variables in Chile**													
(1) Gender (% of male)	0.27	0.45											
(2) Age	31.80	7.57	0.01										
(3) Impression management	5.47	1.39	0.05	0.06									
(4) Materialism at work	2.21	0.53	0.06	-0.05	-0.16^∗∗^								
(5) Basic psychological needs satisfaction	5.63	0.92	-0.04	0.03	0.10^∗∗^	-0.19^∗∗^							
(6) Basic psychological needs frustration	2.58	1.04	-0.05	-0.05	-0.29^∗∗^	0.22^∗∗^	-0.28^∗∗^						
(7) Engagement	5.36	0.76	-0.03	0.16^∗∗^	0.19^∗∗^	-0.12^∗∗^	0.53^∗∗^	-0.30^∗∗^					
(8) Work satisfaction	7.85	1.96	-0.04	0.05	0.17^∗∗^	-0.15^∗∗^	0.51^∗∗^	-0.34^∗∗^	0.63^∗∗^				
(9) Turnover intention	2.14	2.80	0.00	-0.04	-0.14^∗∗^	0.12^∗∗^	-0.27^∗∗^	0.39^∗∗^	-0.32^∗∗^	-0.39^∗∗^			
(10) Burnout	1.64	1.29	0.00	-0.12^∗∗^	-0.25^∗∗^	0.23^∗∗^	-0.35^∗∗^	0.58^∗∗^	-0.48^∗∗^	-0.54^∗∗^	0.45^∗∗^		
(11) Income ($US)	798.84	218.07	0.01	0.19^∗∗^	-0.01	-0.11^∗∗^	0.10^∗∗^	0.07^∗^	0.08^∗^	0.05	-0.01	-0.02	

**Descriptives and inter-correlations between all study variables in Paraguay**													
(1) Gender (% of male)	0.52	0.50											
(2) Age	28.18	5.67	24^∗∗^										
(3) Impression management	4.41	1.63	0.04	0.07									
(4) Materialism at work	2.49	0.40	0.08	-0.01	-0.09^∗^								
(5) Basic psychological needs satisfaction	5.53	0.87	0.04	0.13^∗∗^	0.14^∗∗^	-0.09^∗^							
(6) Basic psychological needs frustration	3.28	1.01	0.07	0.09^∗^	-0.29^∗∗^	0.10^∗^	-0.08						
(7) Job insecurity	4.01	3.02	0.03	-0.01	-0.08	0.07	-0.21^∗∗^	0.28^∗∗^					
(8) Meaning at work	8.42	1.31	-0.04	0.04	0.21^∗∗^	-0.12^∗∗^	0.46^∗∗^	-0.32^∗∗^	-0.20^∗∗^				
(9) Negative emotions at work	5.34	1.64	-0.05	0.01	-0.15^∗∗^	0.19^∗∗^	-0.10^∗^	0.37^∗∗^	0.23^∗∗^	-0.06			
(10) Organizational commitment	4.03	0.52	0.02	0.15^∗∗^	0.23^∗∗^	-0.13^∗∗^	0.39^∗∗^	-0.20^∗∗^	-0.28^∗∗^	0.47^∗∗^	-0.19^∗∗^		
(11) Work satisfaction	8.49	1.83	0.00	0.07	0.16^∗∗^	-0.09^∗^	0.32^∗∗^	-0.24^∗∗^	-0.23^∗∗^	0.52^∗∗^	-0.13^∗∗^	0.51^∗∗^	
(12) Burnout	2.88	1.09	0.03	-0.09^∗^	-0.27^∗∗^	0.14^∗∗^	-0.21^∗∗^	0.51^∗∗^	0.32^∗∗^	-0.40^∗∗^	0.32^∗∗^	-0.47^∗∗^	-0.43^∗∗^

*^∗^*p* < 0.05; ^∗∗^*p* < 0.01.*

## Results

### Chile

**FIGURE 1:**
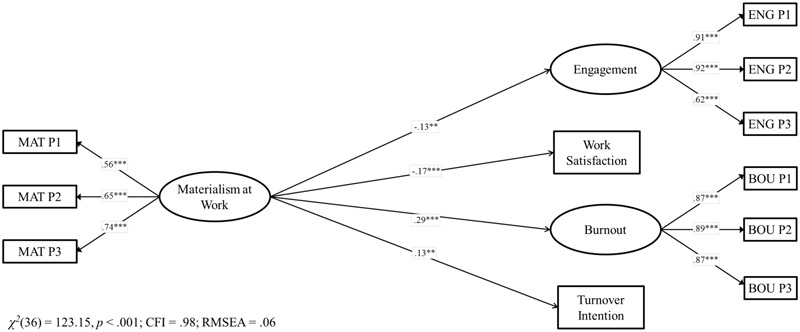
Structural model for the associations between materialism and job outcomes in Chile. Coefficients shown are standardized paths. Error terms and covariances are not shown to enhance visual clarity. Pi, Parcel i; MAT, Materialism; ENG, Engagement; BOU, Burnout. ^∗∗^*p* < 0.01; ^∗∗∗^*p* < 0.001.

**FIGURE 2:**
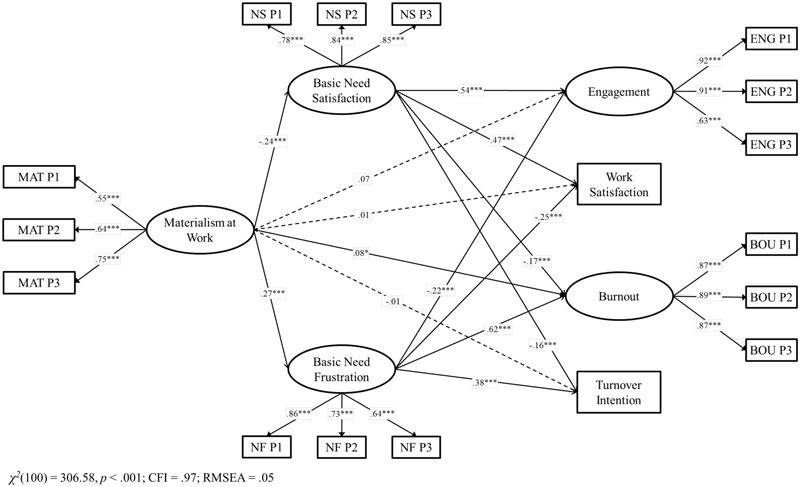
Structural model for the associations between materialism, basic need satisfaction, basic need frustration and job outcomes in Chile. Coefficients shown are standardized paths. Error terms and covariances are not shown to enhance visual clarity. Pi, Parcel i; MAT, Materialism; ENG, Engagement; BOU, Burnout. Solid lines = significant paths; Dashed lines = non-significant paths. ^∗^*p* < 0.05; ^∗∗∗^*p* < 0.001.

Confirmatory factor analysis showed that the expected seven-factor measurement model showed good fit in the Chilean data, χ^2^(100) = 306.58, *p* = <0.001, CFI = 0.97, RMSEA = 0.05, AIC = 412.58. Alternative models such as (a) a single-factor model, χ^2^(44) = 1460.01, *p* < 0.001, CFI = 0.65, RMSEA = 0.21, AIC = 1504.11, (b) a model in which need satisfaction and need frustration were collapsed into a single construct, χ^2^(106) = 1126.27, *p* < 0.001, CFI = 0.85, RMSEA = 0.11, AIC = 1120.27, and (c) a model in which need satisfaction and need frustration as well as the attitudinal and well-being measures were considered as one construct, χ^2^(62) = 1019.09, *p* < 0.001, CFI = 0.75, RMSEA = 0.14, AIC = 1077.09 showed all non-acceptable fits and a higher AIC (Δ_S_ = 1091.53, 707.69, 664.51, respectively) than the original seven-factor model. Therefore, we assumed that our theoretical model is the most likely one^2^. In order to test our first hypothesis about the associations between materialism and employees’ functioning in Chile (Model 1), we created a structural model in which materialism at work predicted positive (engagement and work satisfaction), as well negative (burnout and intention to turnover) outcomes^3^. Model 1 showed a good model fit, χ^2^(36) = 123.15, *p* < 0.001, CFI = 0.98, RMSEA = 0.06 and the explained variance of the constructs ranged from 1.6 to 8.3%, which is consistent with other studies (Unanue et al., 2014). Materialism was associated with lower engagement and work satisfaction as well as with higher burnout and turnover intentions. Therefore, Hypothesis 1 was fully supported in Chile. All standardized β values are reported in **Figures 1, 2**.

**FIGURE 3:**
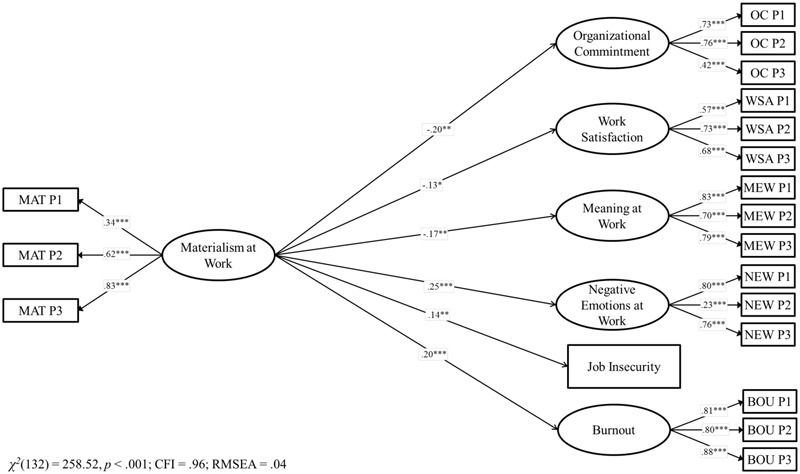
Structural model for the associations between materialism and job outcomes in Paraguay. Coefficients shown are standardized paths. Error terms and covariances are not shown to enhance visual clarity. Pi, Parcel i; MAT, Materialism; MEW, Meaning at Work; NEW, Negative Emotions at Work; OC, Organizational Commitment; WSA, Work Satisfaction; BOU, Burnout. ^∗^*p* < 0.05; ^∗∗^*p* < 0.01; ^∗∗∗^*p* < 0.001.

**FIGURE 4:**
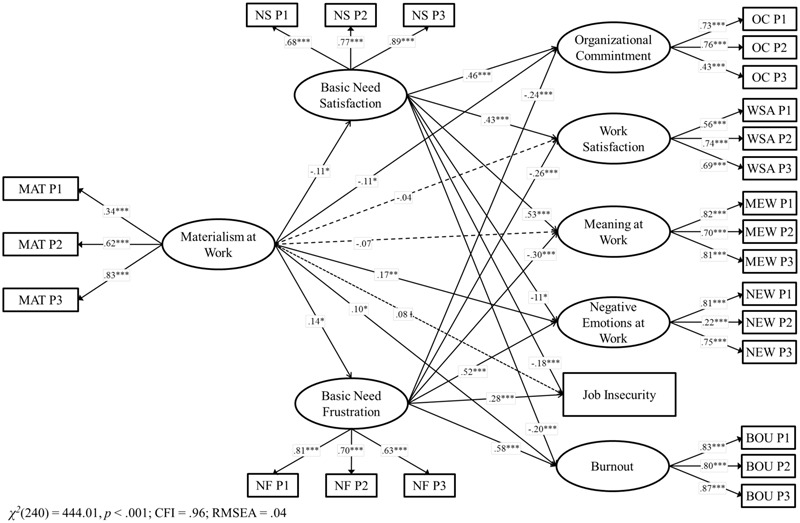
Structural model for the associations between materialism, basic need satisfaction, basic need frustration and job outcomes in Paraguay. Coefficients shown are standardized paths. Error terms and covariances are not shown to enhance visual clarity. Pi, Parcel i; MAT, Materialism; MEW, Meaning at Work; NEW, Negative Emotions at Work; OC, Organizational Commitment; WSA, Work Satisfaction; BOU, Burnout; NS, Need Satisfaction; NF, Need Frustration. Solid lines = significant paths; Dashed lines = non-significant paths; Dotted lines = marginally significant lines. ^†^*p* < 0.10; ^∗^*p* < 0.05; ^∗∗^*p* < 0.01; ^∗∗∗^*p* < 0.001.

To test our second hypothesis, we included both need satisfaction and need frustration as mediators (Model 2) in the relationship between materialism and job outcomes. The model showed a good fit, χ^2^(100) = 306.58, *p* < 0.001, CFI = 0.97, RMSEA = 0.05 and there was also an increase in the explained variance of the constructs in comparison with Model 1. R^2^ ranged from 5.9 to 52.2%. Materialism at work was positively associated with lower need satisfaction, which in turns was associated with higher engagement and work satisfaction as well as with lower burnout and turn over intentions. In addition, materialism was positively associated with higher need frustration, which in turns was associated with lower engagement and work satisfaction as well as with higher burnout and turn over intentions. After including the mediation of both need satisfaction and need frustration together, the direct paths of materialism with the outcomes became non-significant, except for burnout.

Sobel tests confirmed that need satisfaction mediated the relationship between materialism and engagement (*z* = -4.60, *p* < 0.001), work satisfaction (*z* = -4.56, *p* < 0.001), intention to turnover (*z* = 3.10, *p* < 0.05), and burnout (*z* = 3.33, *p* < 0.001), while need frustration also mediated the relationship between materialism and engagement (*z* = -3.82, *p* < 0.001), work satisfaction (*z* = -4.10, *p* < 0.001), intention to turnover (*z* = 4.53, *p* < 0.001), and burnout, *z* = 4.94, *p* < 0.001. Thus, H2 received empirical support in Chile.

Finally, we conducted a formal test of whether need satisfaction and frustration were differentially related to the four job outputs (H3). Following Gausel et al. (2012) and Unanue et al. (2014), we compared Model 2 with an alternative model that constrained the relative weighting of need satisfaction and frustration in predicting the four work outcomes to be equal, although the absolute size of these paths could vary across the four outcomes. We specified a single latent variable (without a disturbance term) as mediating the effects between need satisfaction and need frustration and well-being. The path from need satisfaction to this latent variable was set at 1, whereas the remaining paths were estimated freely. This alternative model showed a significantly worse fit than our original model, Δχ^2^(3) = 134.32, *p* = <0.001, confirming that need satisfaction and frustration were differentially related to the four well-being outcomes. Therefore, H3 was fully supported. We showed that need satisfaction and frustration were differentially related to the four well-being outcomes. Whereas need satisfaction was more strongly related to the positive outcomes, need frustration was more strongly related to the negative ones.

### Paraguay

We followed the same procedure in the Paraguayan data than in the Chilean data to further test the hypotheses. Confirmatory factor analysis showed that the expected nine-factor measurement model showed good fit, χ^2^(240) = 444.01, *p* = <0.001, CFI = 0.96, RMSEA = 0.04, AIC = 614.01. Alternative models such as (a) a single-factor model, χ^2^(152) = 1131.28, *p* < 0.001, CFI = 0.63, RMSEA = 0.12, AIC = 1427.28, (b) a model in which need satisfaction and need frustration were collapsed into a single construct, χ^2^(248) = 1034.501, *p* < 0.001, CFI = 0.83, RMSEA = 0.08, AIC = 1188.50, and (c) a model in which need satisfaction and need frustration as well as the attitudinal and well-being measures were considered as one construct, χ^2^(116) = 884.69, *p* < 0.001, CFI = 0.71, RMSEA = 0.11, AIC = 958.59, showed all non-acceptable fits and a higher AIC (Δ_S_ = 793.27, 574.49, 344.68, respectively) than the original nine-factor model. Therefore, we assumed that our theoretical model is the most likely one.

In testing our first hypothesis, we created a structural model in which materialism at work predicted positive (organizational commitment, work satisfaction and meaning at work), as well negative (negative emotions, job insecurity, and burnout) outcomes (Model 3)^4^. The results showed good model fit, χ^2^(132) = 258.52, *p* < 0.001, CFI = 0.96, RMSEA = 0.04 and the explained variance of the constructs ranged from 1.6 to 6.2%. Materialism was significantly associated with lower organizational commitment, work satisfaction and meaning at work as well as with higher negative emotions, burnout, and job insecurity. Therefore, H1 was fully supported in Paraguay.

To test H2, we included both need satisfaction and frustration together as mediators (Model 4). The model showed good fit, χ^2^(240) = 444.01, *p* < 0.001, CFI = 0.96, RMSEA = 0.04, and there was also an increase in the explained variance of the constructs in comparison with Model 3. R^2^ ranged from 1.2 to 44.1%. Materialism was positively associated with higher need satisfaction, which in turns was associated with higher organizational commitment, work satisfaction, and meaning and work as well as with lower negative emotions, job insecurity, and burnout. In addition, materialism was positively associated with higher need frustration, which in turns was associated with lower organizational commitment, work satisfaction, and meaning at work as well as with higher negative emotions, job insecurity and burnout. After including the mediation of both need satisfaction and need frustration together, the paths between materialism and work satisfaction, meaning at work and job insecurity became non-significant. The other paths remained significant, but its standardized values dropped in magnitude.

Sobel tests confirmed that need satisfaction only marginally mediated the relationship between materialism and organizational commitment (*z* = -1.91, *p* = 0.06), work satisfaction (*z* = -1.87, *p* = 0.06), meaning at work (*z* = -1.93, *p* = 0.05), job insecurity (*z* = 1.76, *p* = 0.08), and burnout (*z* = 1.80, *p* = 0.07). However, it did not mediate the relationship between materialism and negative emotions at work (*z* = 1.45, *p* = 0.15). Need frustration significantly mediated the relationship between materialism and organizational commitment (*z* = -2.02, *p* < 0.05), work satisfaction (*z* = -2.02, *p* < 0.05), meaning at work (*z* = -2.13, *p* < 0.05), negative emotions at work (*z* = 2.20, *p* < 0.05), job insecurity (*z* = 2.11, *p* < 0.05), and burnout (*z* = 2.22, *p* < 0.05), showing the additional powerful explanatory role of need frustration in the Paraguayan context. Thus, Hypothesis 2 received further empirical support.

As in Chile, we conducted a formal test of whether need satisfaction and frustration were differentially related to the four job outputs to test H3. The alternative model showed a significant worse fit than our original model, Δχ^2^(6) = 94.26, *p* = <0.001, confirming that the six outputs were related differently to need satisfaction and need frustration. Thus, our results in Paraguay showed that whereas need satisfaction was more strongly related to the positive outcomes, need frustration was more strongly related to the negative ones. Therefore, Hypothesis 3 was fully supported in Paraguay.

### Supplementary Analyses

Based in SDT, we predicted that materialism would be detrimental across nations and one’s personal income. To further test the generalizability of our results, we tested the moderator effect workers’ incomes on the link between materialism and attitudes/well-being at work in the Chilean data^5^. Following Nickerson et al. (2003), we constructed an interaction term as the product of materialism and income (observed variable). Results showed a good fit, χ^2^(42) = 135.53, *p* < 0.001; CFI = 0.98; RMSEA = 0.06. All the main effects from materialism to engagement, job satisfaction, burnout, and turn over intentions remained significant. Most important, none of the interactions between materialism and income were significant, suggesting that materialism negatively predicted our outcomes variables, regardless of employees’ level of salary, providing further evidence for the detrimental effect of materialism.

Then, to test whether country moderates the relationships between materialism, need satisfaction and need frustration and outcomes, we conducted a multigroup moderator analysis. We only used burnout as an outcome variable, as it is the only common outcome in the two countries.

First, we tested Model 1S. In this model, materialism was allowed to predict burnout. The Model showed a good model fit, χ^2^(16) = 32.92, *p* < 0.001, CFI = 0.99, RMSEA = 0.03. Constraining the path from materialism to burnout to be equal across Chile and Paraguay resulted in a good model fit, χ^2^(17) = 33.25, *p* < 0.001, CFI = 0.99, RMSEA = 0.03, which was not worse than the fit of the model without the path constrained, Δχ^2^(1) = 0.34, *p* = 0.56. Thus, the path between materialism and burnout does not differs significantly between Chile and Paraguay (Chile: β = 0.30, *p* < 0.001; Paraguay: β = 0.18, *p* < 0.001).

Second, we tested Model 2S. In this full model, we allowed materialism to predict burnout, and included both need satisfaction and need frustration as mediators. The model showed a good fit, χ^2^(96) = 216.66, *p* < 0.001, CFI = 0.98, RMSEA = 0.03. Constraining all the paths to be equal across both nations resulted in a good model fit, χ^2^(101) = 222.52, *p* < 0.001, CFI = 0.98, RMSEA = 0.03, which was not worse than the fit of the model without the paths constrained, Δχ^2^(5) = 5.86, *p* = 0.32. Thus, the paths from materialism to burnout (Chile: β = 0.11, *p* < 0.001; Paraguay: β = 0.06, *p* < 0.01), from materialism to need satisfaction (Chile: β = -0.24, *p* < 0.001; Paraguay: β = -0.13, *p* < 0.001), from materialism to need frustration (Chile: β = 0.27, *p* < 0.001; Paraguay: β = 0.13, *p* < 0.001), from need satisfaction to burnout (Chile: β = -0.18, *p* < 0.001; Paraguay: β = -0.18, *p* < 0.001) and from need frustration to burnout (Chile: β = 0.58, *p* <0.001; Paraguay: β = 0.63, *p* < 0.001) do not differs significantly between Chile and Paraguay.

## Discussion

The purpose of this study was to test the link between materialism and employees’ attitudes and well-being, thereby adding to the literature in two ways. First, we aimed to unravel the mechanisms underlying the link between materialism and several desirable and undesirable work outcomes, using both need satisfaction and need frustration as defined in SDT (Deci and Ryan, 2000). Second, we aimed to extend the limited amount of research on the impact of materialistic strivings at work – conducted only in the Western world – exploring for the first time a poorer Latin–American context and taking one’s personal level of income into account. In short, our empirical findings support our hypotheses: need satisfaction and need frustration explain in a unique manner the negative link between materialism and employee attitudes and well-being in poorer countries than studied before and also when taking one’s personal income into account.

More specifically, in two large samples of employee adults from Chile (financial sector) and Paraguay (retail sector) we found that higher materialism at work is associated with less positive and more negative job outcomes. In Chile, employees who highly value materialism are more likely to experience lower engagement and work satisfaction as well as higher turnover intentions and burnout. In Paraguay, we found similar results, but using different measures. We replicated findings regarding work satisfaction and burnout, but additionally, we also found that higher materialism at work is associated with lower organizational commitment, and meaning at work as well with higher negative emotions at work, and job insecurity.

These results extend earlier findings (Vansteenkiste et al., 2007; Deckop et al., 2010, 2015; Schreurs et al., 2014) by showing that the negative relationship between materialism and positive job outcomes and the positive relationship between materialism and negative job outcomes also applies to Latin–American nations. The results seem to contradict strong effects predicted by both the fit-perspective, goal-attainment hypothesis and Maslovian perspectives mentioned above. However, they are congruent with SDT, which argues that the associations may be found across life domains, regardless of countries’ GDP, economic development and other socio-economic indicators. Despite that our paper did not aim to be cross-cultural, our supplementary analyses added preliminary evidence regarding the cross-cultural generalizability of our findings. We showed that the relationships between materialism, need satisfaction and need frustration, and burnout does not differ significantly across countries, which adds to the generalizability of SDT’s assumptions across cultures. Moreover, our analysis (in Chile) showed that materialism impacts negatively workers’ attitudes and well-being, above and beyond the effect of employees’ income.

Our results furthermore show that need satisfaction and need frustration can explain the detrimental impact of materialism: employees who primarily focus on material success, image and recognition (i.e., extrinsic values) instead of personal growth, altruism and developing good relations (i.e., intrinsic values), experienced less basic need satisfaction and higher need frustration at work, which, in turn, led them to have more negative and less positive outcomes at work. Importantly, our results indicate that need frustration plays a key role, in addition to need satisfaction. We thus extended previous research (e.g., Vansteenkiste et al., 2007; Deckop et al., 2015) and established that it is desirable to distinguish between basic need satisfaction and basic need frustration. Our results in both countries showed that it is indeed necessary to model need satisfaction and need frustration as different constructs.

Further, and consistent with recent theorizing (Vansteenkiste and Ryan, 2013) and empirical work (Unanue et al., 2014; Chen et al., 2015; Cordeiro et al., 2016), we showed that whereas need satisfaction related more strongly to positive job outcomes, need frustration did so to negative ones. These results further establish that need satisfaction and frustration are not just each other’s opposite, but divergent constructs. Furthermore, they lend support to the notion that humans have a potential for growth and well-being, but also for malfunctioning and ill-being and that the construct of needs may represent “a major bridge connecting both positive and pathology-focused psychologies” (Vansteenkiste and Ryan, p. 274). In short, to get a deeper understanding of both positive and negative outcomes at work, researchers thus need to take into account both need satisfaction and need frustration and not simply rely on need satisfaction as is done thus far (Van den Broeck et al., 2016).

The discussion about the bipolar dimensionality of positive versus negative side of people’ experiences is not new. For example, in work related well-being contexts, it has already been debated whether people’ positive and negative well-being fit into one and the same general well-being concept or must be differentiated into two separate continua such as ill-being and well-being (Barrett et al., 2007). Our results provide further evidence for the former: we have consistently shown that it is necessary to distinguish between basic need satisfaction (the positive side) and basic need frustration (the negative side), because need satisfaction and need frustration are not simply different ends of the same continua: their interrelations are relatively low and they relate differently to different outcomes. Our results are in line with the meta-analytic findings of Van den Broeck et al. (2016) showing that satisfaction of basic needs does not substantively predict more negative forms of motivation or well-being. Our results even move beyond these findings in that need frustration does relate to these outcomes. In short, need satisfaction is more related to positive forms of motivation and well-being, while need frustration is more related to negative ones. Importantly in view of DT, autonomy, competence, and relatedness are thus not only a source of growing and well-being, they could also be a source of misery and suffering when they are thwarted. More research on need frustration is needed, particularly studies including both need satisfaction and need frustration. Such studies could answer questions such as: what are the underlying psychological mechanisms and process behind basic need satisfaction and basic need frustration? What are the different consequences of need satisfaction and need frustration? This would be highly relevant, also in the occupational context, where job satisfaction and dissatisfaction emerge from different processes that hold different antecedents and consequences (Barrett et al., 2007).

### Limitations and Future Research

Like any study, we should acknowledge some limitations of our research. First, all our measures are self-reported, increasing the risk of common-method bias. However, self-reports measures are justifiable when studying constructs that are self-referential, such as our constructs of need satisfaction, engagement and burnout (Chan, 2009). In addition, we took several *a priori* precautions (Conway and Lance, 2010) in order to mitigate common-method bias: controlling for response bias (e.g., impression management); using of construct-valid measurement scales; protecting respondent anonymity, instructing that there were no right or wrong answers (Podsakoff et al., 2003) and allowing participation at the location of employees choosing (e.g., home, office, etc.). Finally, results from our single-factor measurement models showed that our results are not likely affected for method bias (Landry et al., 2016). Nonetheless, future research could consider using both subjective and objective measures for example in assessing other outcomes such as physical health or performance, extending our results to employee’s behaviors, taking a two-approach data collection, and asking “significant others” to report on the participants’ attitudes and well-being.

Second, single item measures might be some time problematic. We used single items measures for work satisfaction as well as for employee’s intention to turnover and job insecurity. However, previous research and meta-analytic analysis demonstrated that the work satisfaction item we used in the current research has good psychometric properties, and therefore could be used without problems (Wanous et al., 1997; Dolbier et al., 2005). In addition, meta-analytic findings have shown that single-item questions – as the one we used to measure turn over intentions – are a comparatively good way to assess the construct (Tett and Meyer, 1993). Finally, *job insecurity* was measured asking participants to what extend they think they *“could be fired”* during the following months. This question may bias the response, because it includes only the state “want to quit.” Further research should also include elementary contra-balance items (e.g., “To what extent have you want to remain in your organization?”).

Third, our correlational design does not allow us to infer causality. Does materialism lead to lower well-being at work or does lower well-being at work lead to higher materialism at work? Future research should complement our findings by using either experimental or longitudinal data to disentangle the causal direction. Nonetheless, our proposed model falls in line with previous research (see Dittmar et al., 2014) and adds important information to the literature on materialism from a SDT perspective in the organizational arena. Importantly, by testing alternative models, we showed that our proposed sequence is the more likely than any other (results available upon request).

Fourth, we should acknowledge that the effects sizes of basic needs on work outcomes were substantially larger than the effect sizes of materialism on work outcomes. While need satisfaction/frustration are important factors in predicting work outcomes, materialism is thus only one among many variables influencing employees’ attitudes and well-being (Unanue et al., 2014). The effected sizes of materialism reported here are in the range shown by Dittmar et al.’s (2014) meta-analysis. Interestingly, materialism is generally more strongly linked to negative (e.g., burnout) than to positive outcomes (e.g., engagement). The study of materialism thus becomes even more relevant when exploring maladaptive employee functioning and work-related problems.

Fifth, we acknowledge that the selection of our samples from two different companies in two different countries may introduce some bias. Indeed, the samples from Chile and Paraguay may not be representative for all employees in these countries nor the country populations. Surprisingly little is known about the cultural values in Chile and Paraguay from renowned studies on cultural values such as the study of Hofstede and Bond (1984) or the Globe project (Dorfman et al., 2012). First insights suggest that some differences with other parts in the world exist. For example, people in Chile seem to attach more value to power distance and uncertainty avoidance compared to people in the United States, while also highly valuing collectivism and feminine values^6^. From research examining the impact of socio-economic and cultural processes on people’s values in the United States (Twenge and Kasser, 2013), it may be further deduced that people growing up in poorer circumstance may attach higher importance to materialistic values. However, exactly which cultural values prevail in Chile and Paraguay remains to be studied as is the link between cultural values and people’s life and work values (Roe and Ester, 1999). We thus have to be careful of generalizing the current findings to their respective cultures. Nonetheless, our results highly strength the generalizability of the link between materialism, need satisfaction/frustration and job attitudes and well-being across samples of different nations.

Sixth, we controlled for age, gender, and social desirability. Nonetheless, future research may explore also the impact of other different demographic variables such as job tenure, marital status, educational level, number of dependents, and so on.

Seven, and finally, further research should explore the relationship between materialism and burnout in more detail. Even after the inclusion of both need satisfaction and need frustration as mediators, the direct path between materialism and burnout remained significant in both samples. This indicates that there are other underlying causes and explanatory variables for this ill-being indicator. Given the high incidence of work-related stress nowadays, better understanding of burnout and its relationship with materialism may help to improve employees and countries quality of life.

### Practical Contributions

In studying materialism, our research also has practical implications. Given the effect that materialism has on engagement (and other job outcomes) which in turn lead to significant improvements in company revenues, sales and profits (Harter et al., 2010), our research advances that companies would do well-promoting an intrinsic rather than extrinsic mind-set among their employees, as promoting intrinsic values might strength the link between employees and the organization, providing the companies with “a unique supportive climate that employees may not find elsewhere” (Van den Broeck et al., 2014, p. 1913). Although many different aspects ranging from cultural processes to one’s general life values may influence one’s personal work values (e.g., Roe and Ester, 1999), organizations can also aim to decrease materialism among employees by relying on intrinsic (e.g., job design feedback) instead of extrinsic (e.g., bonus culture) management systems. For example, whereas nowadays reward systems frequently emphasize financial success and material possessions as means of motivation and control (Bates, 2003), companies could re-design them carefully and include for example also intrinsic aspects (e.g., health insurance) in the total benefits plans to increase their intrinsic value.

Notably, basic need satisfaction and frustration play a mediating role in the relationship between materialism and attitudes and well-being, and are strong predictors of the latter. Our results thus further support that need satisfaction is a promising mechanism through which organizational policies can be adjusted. Meta-analytical results for example suggest that organizations may aim to foster the basic needs and limit need frustration by stimulating transformational leadership, justice and providing high quality work, while limiting for example organizational politics (Van den Broeck et al., 2016). Our findings may help scholars, but also practitioners to explore different ways to increase people’s quality of life, but also to reduce people’s suffering and misery in the workplace.

## Conclusion

Based on the previous findings, the general conclusion from this study is that organizations should encourage employees to pursue intrinsic values (e.g., self-development, affiliation, and community involvement) instead of stimulating extrinsic values (e.g., financial success, fame, and image) in order to improve employees well-being and positive attitudes toward work. Indeed, organizations should constantly and critically assess their cultures, and reward systems in order to prevent the negative effects of materialistic values on employees’ well-being, and – hence – the company’ results (Deckop et al., 2015). In this process, the satisfaction and frustration of our basic psychological needs played a key role. As such, the current study contributes to human resources management practices and SDT by highlighting the importance of values on employees’ well-being and companies’ profitability and sustainability.

## Author Contributions

All authors listed have made substantial, direct and intellectual contribution to the present paper. WU, KR, MG, and AVdB wrote several sections of the initial draft, carried out the analyses, interpreted the results and discussed the implications. All authors wrote, read and revised the final paper and approved it for submission at Frontiers in Psychology. This project was also part of KR’s M.Sc. thesis at Universidad Adolfo Ibáñez Business School.

## Conflict of Interest Statement

The authors declare that the research was conducted in the absence of any commercial or financial relationships that could be construed as a potential conflict of interest.

## Footnotes

1 Several absolute and relative measures have been proposed to assess materialism. However, our decision to use a relative measure was influenced by previous research. For example, Dittmar et al. (2014) meta-analysis found that whereas relative measures capture the full meaning of the construct, absolute measures do not. In fact, any particular goal or value exists within a broader system of values and goals and thus, optimal assessment should involve measuring the importance of a particular goal (e.g., extrinsic goals), relative to other goals in that system (e.g., intrinsic goals). Further, Dittmar et al. (2014) strongly recommended that researchers in the field should employ relative measures such the aspiration index. Therefore, we followed Dittmar et al. (2014) recommendation.

2 We used G^∗^Power 3.1 (Faul et al., 2009) to run a statistical test of power, following Cohen (1992) criteria. To be conservative, we hypothesized an effect size (ES) = 0.15 and an alpha value of 0.05. Our results showed that the required sample size under these parameters is *N* = 43 for a power of 0.80. Therefore, we concluded that our current samples size (*N* = 742) fulfills this requirement. It is consistent with recent recommendations based on Monte Carlo research which has shown that as few as 30 or as many as 460 participants are enough for popular analyses (Wolf et al., 2013). Data from Paraguay also fulfill sample sizes requirements.

3 In testing all our hypotheses, we allowed our outcomes variables to covary (Kline, 2011). First, we controlled for age, gender, and social desirability. However, none of these control variables significantly changed the relationship between the variables. The only difference we found between the model without control variables and the one with them, is that the path between materialism and engagement become marginally significant in the later. Hence, for simplicity, we reported all our further analyses without control variables.

4 As in Chile, we first controlled for age, gender, and social desirability. However, none of these control variables significantly changed the relationship between the variables. The only difference we found between both models is that the path between materialism and work satisfaction become marginally significant when using control variables. Hence, for simplicity, we reported all our further analyses without them. All standardized β values are reported in **Figures 3** and **4**.

5 It was not possible to test H2 in Paraguay, because we did not collect objective measures of income in this country.

6 https://geert-hofstede.com
